# Identification and Validation of a Hypoxia-Immune-Based Prognostic mRNA Signature for Oral Squamous Cell Carcinoma

**DOI:** 10.1155/2022/5286251

**Published:** 2022-02-07

**Authors:** Shaohua Lv, Zhipeng Qian, Jianhao Li, Songlin Piao, Jichen Li

**Affiliations:** ^1^Department of Oral and Maxillofacial Surgery, The First Affiliated Hospital of Harbin Medical University, Harbin 150081, China; ^2^Stomatology School, Harbin Medical University, 143 Yiman Street, Nangang District, Harbin, Heilongjiang, China; ^3^College of Bioinformatics Science and Technology, Harbin Medical University, Harbin, China

## Abstract

**Background:**

Oral squamous cell carcinoma (OSCC) is a commonly encountered head and neck malignancy. Increasing evidence shows that there are abnormal immune response and chronic cell hypoxia in the development of OSCC. However, there is a lack of a reliable hypoxia-immune-based gene signature that may serve to accurately prognosticate OSCC.

**Methods:**

The mRNA expression data of OSCC patients were extracted from the TCGA and GEO databases. Hypoxia status was identified using the t-distributed Stochastic Neighbor Embedding (t-SNE) algorithm. Both ESTIMATE and single-sample gene-set enrichment analysis (ssGSEA) were used for further evaluation of immune status. The DEGs in different hypoxia and immune status were determined, and univariate Cox regression was used to identify significantly prognostic genes. A machine learning method, least absolute shrinkage and selection operator (LASSO) Cox regression analysis, allowed us to construct prognostic gene signature to predict the overall survival (OS) of OSCC patients.

**Results:**

A total of 773 DEGs were identified between hypoxia high and low groups. According to immune cell infiltration, patients were divided into immune high, medium, and low groups and immune-associated DEGs were identified. A total of 193 overlapped DEGs in both immune and hypoxia status were identified. With the univariate and LASSO Cox regression model, eight signature mRNAs (FAM122C, RNF157, RANBP17, SOWAHA, KIAA1211, RIPPLY2, INSL3, and DNAH1) were selected for further calculation of their respective risk scores. The risk score showed a significant association with age and perineural and lymphovascular invasion. In the GEO validation cohort, a better OS was observed in patients from the low-risk group in comparison with those in the high-risk group. High-risk patients also demonstrated different immune infiltration characteristics from the low-risk group and the low-risk group showed potentially better immunotherapy efficacy in contrast to high-risk ones.

**Conclusion:**

The hypoxia-immune-based gene signature has prognostic potential in OSCC.

## 1. Introduction

The incidence of lip and oral cavity cancers has been on the rise, with an estimated 246,420 cases and 119,693 deaths occurring globally in 2018 [1]. Oral squamous cell carcinoma (OSCC) is the dominating subtype of oral cancer, which unfortunately harbors a dismal 5-year survival rate [2]. There is still an urgent need to discover accurate prognostic biomarkers and effective drug targets for OSCC.

Solid tumors such as OSCC are usually characterized by cellular hypoxia, which is generally considered to be a manifestation of poor prognosis [3, 4]. Cell hypoxia has evolved to represent a significant feature in cancer [5]. Hypoxic conditions could trigger the migration and invasion of OSCC cells [6–8]. Reports also suggest that hypoxia can induce epithelial to mesenchymal transition (EMT) in several types of cancer, including OSCC [9]. It can also stimulate angiogenesis through the activation of proangiogenic factors [10]. Zhang et al. reported that the expression of HIF-1*α*, Glut-1, and CA9 can predict malignant transformation to OSCC [11]. It is undeniable that cellular hypoxia contributes significantly to the malignant properties of OSCC.

The immune system is crucial in OSCC tumorigenesis, progression, and prognosis [12–14]. Enhanced PD-1 and PD-L1 expressions were observed in OSCC patient serum and tissue samples [15]. Premalignant lesions were also found to harbor raised PD-L1 expressions [16]. Inhibiting the PD-1/PD-L1 axis is one of the most promising means to activate antitumor immunity [17]. A number of immune-related prognostic biomarkers have been identified in OSCC. Meehan et al. had previously constructed an immune-related signature that correlated with OSCC patient prognosis [18]. Some immune checkpoints have been documented to be closely related to poorer patient outcomes in OSCC [19]. One of them is the CTSG gene, which is an immune-related gene and has been identified as an independent biomarker and therapeutic target of OSCC [20]. Although these immune-related prognostic biomarkers have been discovered, few studies have explored the potential benefit of a combined assessment of both immunological characteristics and the hypoxic microenvironment in OSCC [21].

Several studies have alluded to the clinical significance of potential interactions between immune function and cellular hypoxia across various malignancies [22, 23]. Computational framework for prognosis assessment for cancer patients has been widely used in various tumors [24–27]. Although some hypoxia-related and immune-related models have been constructed to predict the prognosis of OSCC [28, 29], a reliable hypoxia-immune-integrated prognostic gene signature has not yet been established for OSCC. Here, we first accessed the mRNA expression profiles of OSCC patients from the TCGA database. Cell hypoxia was identified using the t-SNE algorithm, while the immune status and immune-related DEGs were identified using the ESTIMATE and ssGSEA algorithms. DEGs that were significant in both immune and cellular hypoxia phenomena were identified. This pool of genes was then subjected to a machine learning assessment to discern those which were potentially prognostic. We successfully constructed a hypoxia-immune-based gene signature that was useful in predicting the overall survival (OS) of OSCC patients.

## 2. Materials and Methods

### 2.1. Data Collection and Preprocessing

The discovery cohort included 303 OSCC individual data from the “TCGA-HNSC” project in The Cancer Genome Atlas (TCGA). The Genomic Data Commons (available at https://portal.gdc.cancer.gov) database was then accessed to extract the corresponding gene expression data. In this study, samples without data on the survival state and survival time were eliminated. Data were externally validated using an independent cohort. The expression profile of GSE41613 was downloaded from the Gene Expression Omnibus (http://www.ncbi.nlm.nih.gov/geo/) based on the GPL570 Affymetrix Human Genome U133 Plus 2.0 Array platform. A total of 97 OSCC patient data were included in this validated study. The IMvigor210 (*n* = 348) cohort with immunotherapy data and corresponding clinicopathological information was obtained from the IMvigor210CoreBiologies R package.

RNA-sequencing data (FPKM values) extracted from the TCGA database were translated into transcripts per kilobase million (TPM) values and normalized using log2 (TPM + 1). Raw GEO database-derived data were obtained using the RMA algorithm. Background adjustment, quantile normalization, and final summation of oligonucleotides per transcript were carried out using the median Polish algorithm of the Affy software package. Finally, the different probe IDs were converted into their respective gene symbols to annotate them, and the repeated gene expression values were averaged. All patients with included clinical information and survival data were also incorporated in this study.

### 2.2. Identification of Hypoxia Status and Hypoxia-Related Differentially Expressed Genes (DEGs)

The presence of cell hypoxia was determined using the t-distributed Stochastic Neighbor Embedding (t-SNE) algorithm. This nonparametric, unsupervised method was able to distinguish between patient clusters based on provided hallmarks or signatures. Based on the seven hypoxic signatures of Buffa, Elvidge, Eustace, Hu, Ragnum, Sorensen, and Winter [30], a nonlinear dimensionality reduction algorithm t-SNE was used to measure the Euclidean distance of any two patients in the TCGA cohort, which was then condensed into two-dimensional points. Three clusters were identified. Moreover, we obtained genes using the KEGG HIF-1 signaling pathway (https://www.kegg.jp/; ID:04066) to assess the hypoxic status. Of these genes, 14 were identified as “increased oxygen delivery” genes and 12 were identified as “reduced oxygen consumption” genes. Finally, the “DESeq2” software package was used to discern between DEGs significant to low- or high-hypoxic groups (*P*-value <0.05 and ǀ log2 (fold change) ǀ>1).

### 2.3. Identification of Immune Status and Immune-Related DEGs

We evaluated enrichment degrees of a total of 28 immune cells within each TCGA OSCC sample using the ssGSEA method. The OSCC samples were arbitrarily divided into high-, medium-, and low-immune groups using hierarchical clusters (namely, “Immune_Low,” “Immune_Medium,” and “Immune_High”) based on the above immune matrix. Both degrees of immunocyte infiltration degree (immune score) and stromal level (stromal score) were assessed using the Estimation of Stromal and Immune cells in Malignant Tumor tissues using Expression data (ESTIMATE) algorithm in TCGA OSCC samples to validate the above immune status grouping. The immune-related DEGs between the Immune_Low group and the Immune_High group were identified by the “DESeq2” software package using similar parameters as in the cell hypoxia analysis. The Metascape (https://metascape.org/) database was used to construct GO and KEGG enrichment pathways of the selected DEGs.

### 2.4. Construction and Verification of Prognostic Signatures Related to Hypoxia and Immune Status

We took the intersection between hypoxia- and immune-related differentially expressed genes (DEGs) and selected those overlapped genes for subsequent analysis. First, using the “survival” package in R, we employed univariate Cox regression on these overlapped genes and overall survival (OS) of OSCC in the TCGA to identify survival-related hypoxia-immune-related DEGs. Those with a *P* < 0.05 were considered as significant. Then, using the “glmnet” package in R, the Least Absolute Shrinkage and Selection Operator (LASSO) Cox regression model was applied to select the optimal variables from all identified hypoxia-immune-related prognostic DEGs in the discovery cohort. LASSO is a kind of linear regression that uses shrinkage and can be applied to high-dimensional data. In this study, fivefold cross-validation was employed to select the minimal penalty term (*λ*). All patients were then subjected to a risk score calculation as follows:

risk score = ∑ (coefficient *∗* expression of the signature gene).

The risk score was defined as the sum of the product of the prognostic signature gene expression levels and the corresponding LASSO model-derived coefficients. The optimal cut point was determined by a method of maximally selected rank statistics based on an individual level risk score to further refine a means to stratify patients with OSCC according to their prognosis.

### 2.5. Relationship between Risk Scores and the Immune Microenvironment of OSCC

Profiles of 22 immune cell infiltration were determined in both the low- and high-risk groups using the CIBERSORT algorithm. Using gene expression data, the CIBERSORT is a deconvolution algorithm specifically designed to evaluate cellular composition in tissues. In addition, two immune checkpoints (CTLA-4 and PD-1), APM, CYT, TILs, and TIS allowed for further analysis of the relationship between the low- and high-risk groups.

The major histocompatibility complex (MHC) molecules were subjected to relative antigen presentation mechanism (APM) calculation. The degree of immune cytotoxic activity (CYT) based on granzyme A (GZMA) and perforin-1 (PRF1), both of which are related to CD8 + T cell activation, was calculated on previously reported formulas. Tumor prognosis is known to be associated with the density of T cells tumor infiltration. The proportion of tumor-infiltrating lymphocytes (TILs) was calculated. Lastly, we used the tumor inflammation signature (TIS), which represents a commonly used 18-gene signature in research, to quantify background adaptive immune responses, which are normally inhibited in tumors. The TIS score was derived in terms of the mean of log2-FPKM gene expression of the selected marker genes.

### 2.6. Statistical Analysis

The R version 3.6.1 and its related packages were used to carry out all data analyses. The t-SNE algorithm was performed using the “Rtsne” of the R package on the basis of nonlinear dimensionality reduction. The “estimate” package was used to determine immune scores. The “glmnet” package was used to carry out LASSO Cox regression modeling. To identify independent risk factors for survival, univariate and multivariate Cox regression analyses were employed after the adjustment of covariates. The “survival” package was used to perform the Cox regression model and Kaplan–Meier analyses. A *P* value of <0.05 suggested that the results were statistically significant.

## 3. Results

### 3.1. Hypoxia Status and Hypoxia-Related DEGs in OSCC

**Figure 1:**
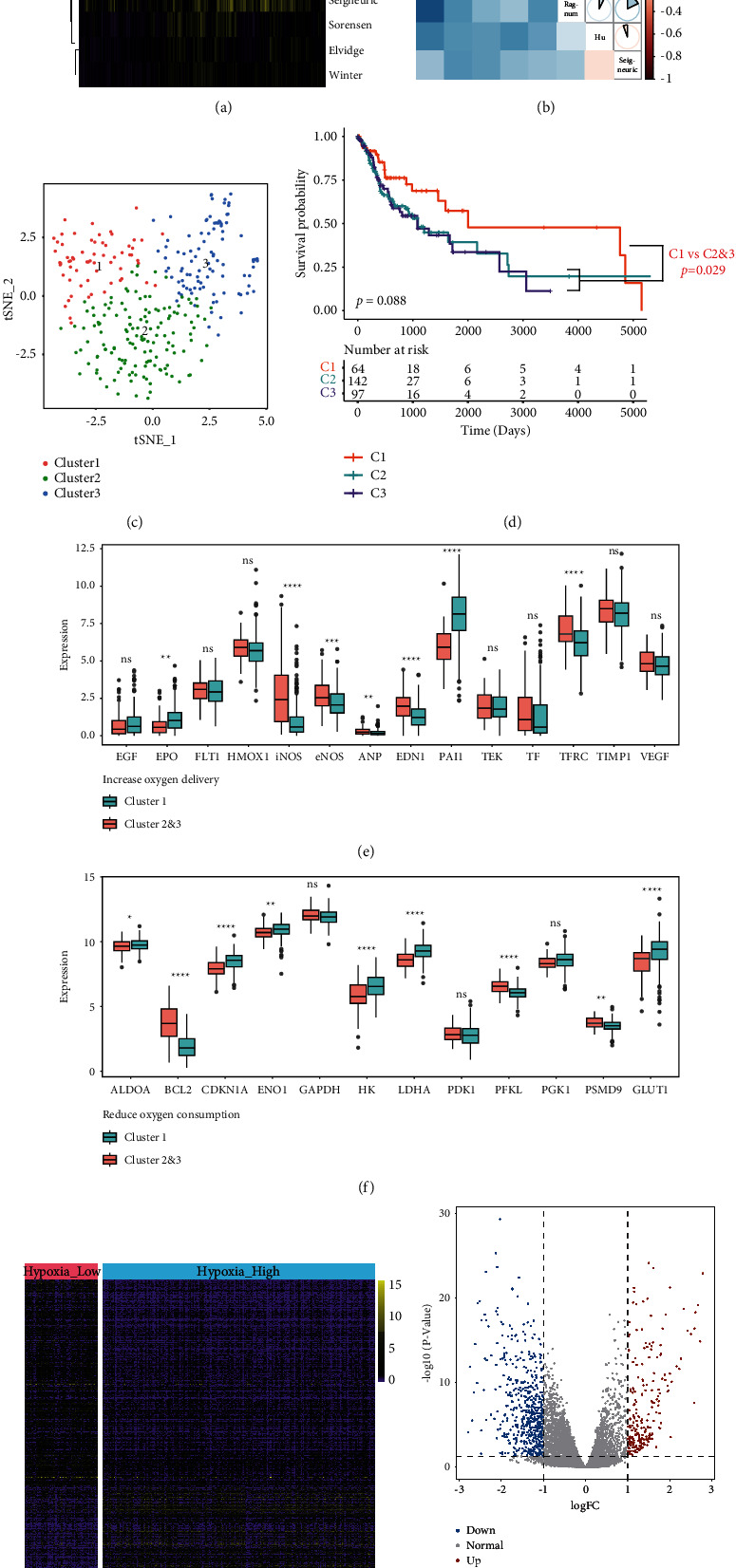
Identification of hypoxia status and hypoxia-associated DEGs. Quantified heatmap (a) and correlation heatmap (b) calculated by ssGSEA with respect to the eight hypoxia signature sets. (c) Dot plot of three distinct clusters determined using the t-SNE algorithm to analyze 7 hypoxia signature gene sets. (d) Overall survival as shown by the Kaplan–Meier plot for patients in three clusters. (e), (f) HIF-1 KEGG pathway gene expression changes based on hypoxia status (Hypoxia_High versus Hypoxia_Low). Heatmap (g) and volcano plot (h) show the differentially expressed hypoxia-related genes in oral squamous cell carcinoma (OSCC). Red dots represent upregulated DEGs, blue dots represent downregulated DEGs, and gray dots represent genes with no differential expression.

In the TCGA cohort, cell hypoxia status was evaluated using ssGSEA analysis based on eight hypoxia signatures (Buffa, Elvidge, Eustace, Hu, Ragnum, Seigneuric, Sorensen, and Winter) [30] (Figure 1(a)). Based on the quantitative score of each hypoxia signature, we found that the “Seigneuric” signature had the lowest correlation with the others and was therefore not included in this study (Figure 1(b)). The remaining seven gene signatures were used in the nonlinear dimensionality reduction algorithm calculation of two-dimensional points between any two patients (see Methods for details). As shown in Figure 1(c), three clusters of patients were identified, and each patient was assigned to their nearest cluster. A total of 64, 142, and 97 cases were classified into Cluster 1, Cluster 2, and Cluster 3. There was no significant difference in terms of patient survival among the three clusters (log-rank test, *P*=0.088) (Figure 1(d)). However, there was a significant difference when comparing Cluster 1 with Cluster 2 and Cluster 3 (log-rank test, *P*=0.029). Clusters 2 and 3 were then combined for further comparison against Cluster 1, which appeared to possess the most favorable overall survival outcome. Clusters 2 and 3 may differ from Cluster 1 in terms of the degree of cellular hypoxia. We further explored differences in expressions of KEGG HIF-1 pathway molecules between the two new clusters (Cluster 1 versus Clusters 2 and 3). The resultant identified genes were classified as being involved in “reduced oxygen consumption” (12 genes) or “increased oxygen delivery” (14 genes). Of those 14 genes involved in increased oxygen delivery, 11 (78.57%) were found to be overexpressed in Clusters 2 and 3 compared with those in Cluster 1 (Figure 1(e)). Seven of the 12 genes related to reduced oxygen consumption (58.33%) were noted to be overexpressed in Clusters 2 and 3 (Figure 1(f)). We therefore proved that both these clusters were of different cell hypoxia status. Patients in Clusters 2 and 3 and Cluster 1 were then renamed to Hypoxia_High or Hypoxia_Low groups, respectively. A total of 773 DEGs were then identified using the DESeq2 package between the Hypoxia_High and Hypoxia_Low groups. Of these genes, 220 were upregulated, whereas 553 were downregulated (Figures 1(g) and 1(h)).

### 3.2. Immune Status and Immune-Related DEGs in OSCC

**Figure 2:**
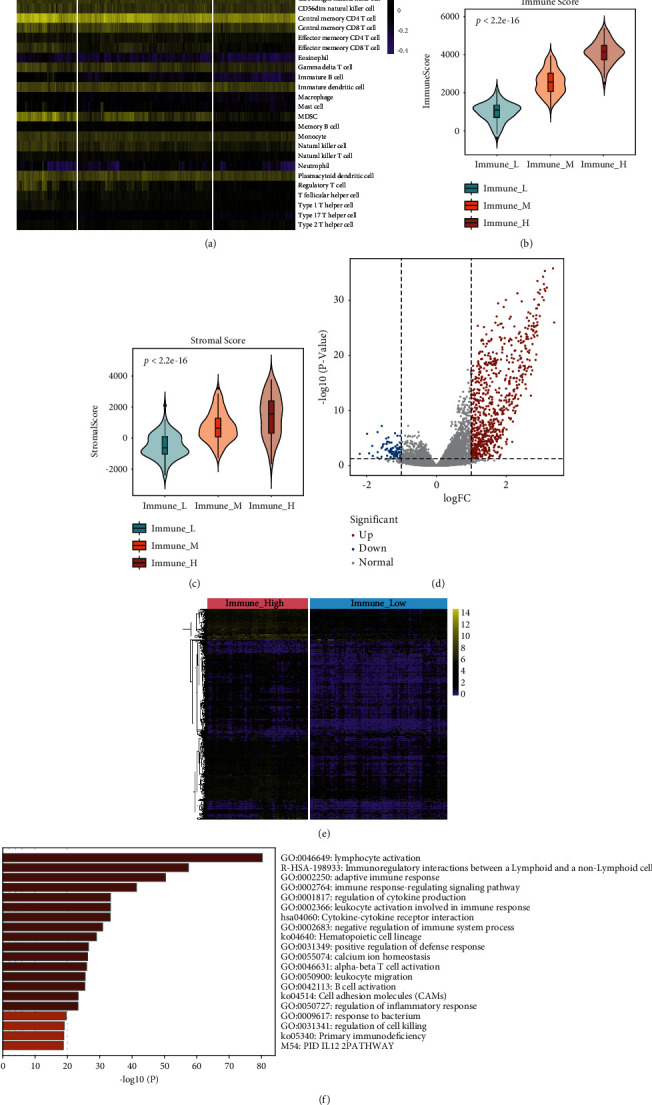
Identification of immune status and immune-associated DEGs. (a) Data from the TCGA database were classified into three subtypes (Immune_High group, Immune_Medium group, and Immune_Low group) based on immune status. Comparison of Immune_High and Immune_Low groups in terms of (b) immune score and (c) stromal score. (d) Volcano plot and (e) heatmap depicting DEGs between Immune_High and Immune_Low groups. Red dots represent upregulated DEGs, blue dots represent downregulated DEGs, and gray dots represent genes with no differential expression. Bar plot (f) of functional enrichment analysis of immune-related DEGs.

Based on the proportions of 28 immune cells quantified by ssGSEA, OSCC samples in the TCGA database were classified into high-, medium-, and low-immune groups (named Immune_ High, Immune_ Medium and Immune_ Low, resp. (Figure 2(a)). In accordance with calculations using the ESTIMATE approach, both immune and stromal scores of the Immune_ High group were elevated in contrast to those of the Immune_ Low group (Figures 2(b) and 2(c)). Comparisons between high- and low-immune groups revealed 854 immune-related DEGs (Figures 2(d) and 2(e)), which were subjected to Metascape tool functional enrichment analysis. The identified DEGs were predominately involved in immune response processes such as “lymphocyte activation,” “adaptive immune response,” “leukocyte activation involved immune response,” and “immunoregulatory interactions between a Lymphoid and a non-Lymphoid cell” (Figure 2(f)).

### 3.3. Development and Validation of the Risk Score

**Figure 3:**
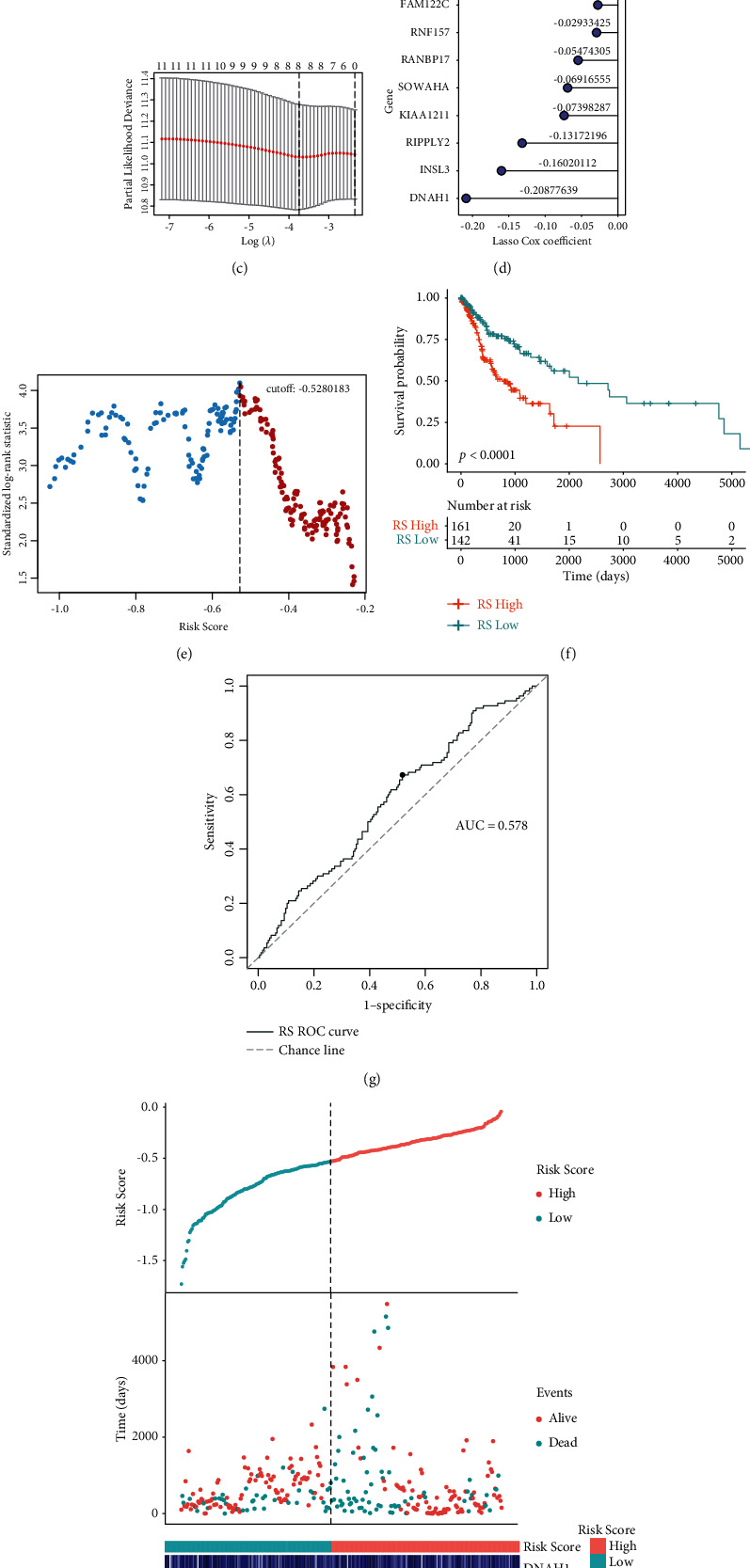
The construction of hypoxia-immune-based mRNA prognosis model. (a) Hazard ratios for 11 hypoxia-immune-associated prognostic mRNAs. (b) LASSO coefficient profiles. (c) The LASSO model is used to choose the tuning parameter (lambda) using fivefold cross-validation based on minimum criteria for OS; average OS genes are represented by the upper *x*-axis, and log (lambda) is represented by the lower *x*-axis. Partial likelihood deviance error is represented by the *y*-axis. (d) An ensemble of 8 genes with their respective individual coefficients. (e) Scatter plot illustrates standardized log-rank statistic value for each corresponding cutoff of hypoxia-immune-based risk score. The optimal cutoff with the maximum standard log-rank statistic is marked with a vertical dashed line. (f) Kaplan–Meier plot of overall survival for patients in low-risk and high-risk groups by hypoxia-immune-based prognosis classifier in the discovery cohort. (g) The effectiveness of the risk scores is predicted using the ROC curve. (h) Expression profiles, survival status, and risk score distributions of the gene signature.

A total of 193 mRNAs were significant in both hypoxia- and immune-related genes. Eleven of these mRNAs were associated with OS (*P* < 0.05) according to univariate Cox regression analysis (Figure 3(a)). Of these, a total of 8 mRNA signatures were selected by the LASSO Cox regression model (Figures 3(b) and 3(c)). All the 8 mRNAs were protective against OSCC (Figure 3(d)). Subsequent risk scores were derived from the following equation: risk score = [DNAH1 expression ^*∗*^ (−0.20877639)] + [FAM122C expression ^*∗*^ (−0.02762894)] + [INSL3 expression ^*∗*^ (−0.16020112)] + [KIAA1211 expression ^*∗*^ (−0.07398287)] + [RANBP17 expression ^*∗*^ (−0.05474305)] + [RIPPLY2 expression ^*∗*^ (−0.13172196)] + [RNF157 expression ^*∗*^ (−0.02933425)] + [SOWAHA expression ^*∗*^ (−0.06916555)]. Using this formula, patients were stratified into either low- or high-risk groups. The method of maximally selected rank statistics was used to determine the optimal risk score cutoff (Figure 3(e)). Significantly improved survival was observed in those in the low-risk group compared to their high-risk counterparts (log-rank test, *p* < 0.001) (Figure 3(f)). The ROC curve showed that the classifier had relatively strong predictive power in the TCGA cohort, with a value of 0.578 for the area under the curve (AUC) (Figure 3(g)). Furthermore, the TCGA cohort was also analyzed for distribution of selected gene expressions and risk scores as well as survival status (Figure 3(h)).  Table 1 depicts all relevant clinical parameters.

**Figure 4:**
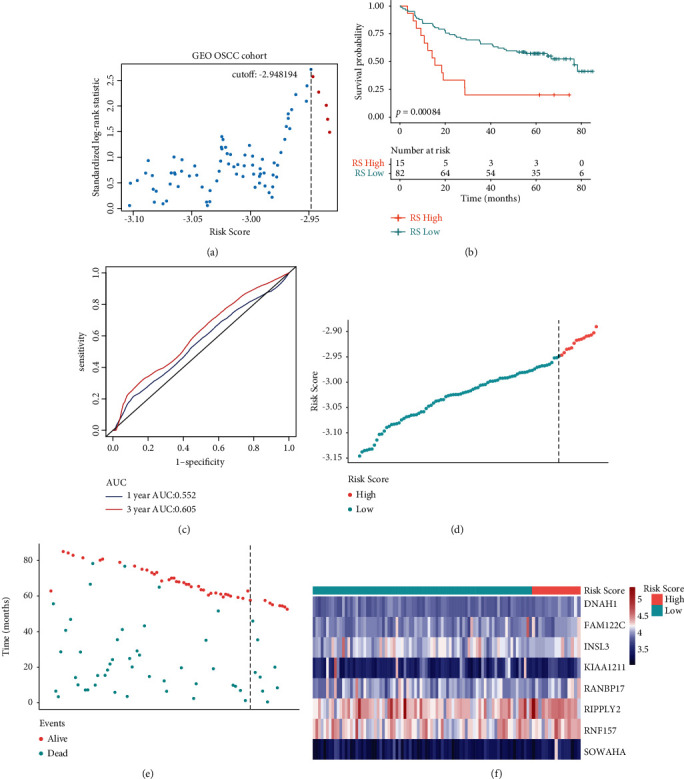
The GEO cohort was used to verify the hypoxia-immune-based prognosis classifier. (a) The scatter plot demonstrates the standardized log-rank statistic value for each corresponding cutoff of hypoxia-immune-based risk score. Vertical dashed lines represent the optimal cutoff with the maximum standard log-rank statistic. (b) The hypoxia-immune-based prognosis classifier is used to label patients from the GEO cohort as either low or high risk, followed by the construction of an overall survival Kaplan–Meier plot. (c) One-year and 3-year survival rates of OSCC patients in the GEO cohort as shown using ROC curves. (d) Distributions of risk score, (e) survival status, and (f) expression profile of signature genes.

To further explore the efficacy of the constructed risk score, we performed validation tests using the GEO cohort. Using the method of maximally selected rank statistics, all OSCC patients in the GEO cohort were divided (Figure 4(a)). Comparison of survival showed that improved survival rates were experienced by low-risk patients in contrast to high-risk patients (log-rank test, *p*=0.00084) (Figure 4(b)). In addition, we plotted the ROC curves for predicting one- and three-year survival according to the OS of patients, and the values of the area under the curve (AUC) were 0.552 and 0.605, respectively (Figure 4(c)). The GEO OSCC cohort was then analyzed for distribution of selected gene expressions and risk scores as well as survival status (Figures 4(d)–4(f)). Furthermore, we investigate the expression difference of eight signature genes between high and low groups. The result showed that the expression of the eight signature genes was significantly higher in the low-risk group than in the high-risk group in both TCGA and GEO cohorts (TCGA: Supplementary Figure 1A; GEO: Supplementary Figure 1B).

### 3.4. Relationship between Prognostic mRNA Signature and Clinical Parameters

**Figure 5:**
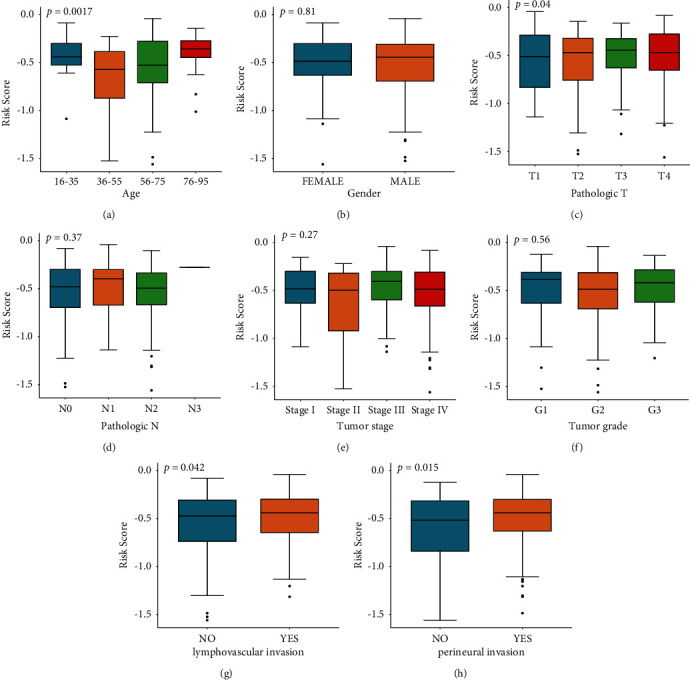
The TCGA cohort was used to determine the association between clinical parameters and the gene signature. Significant association was only found with (a) age, but not with (b) gender, (c) T stage, (d) N stage, (e) tumor stage, (f) tumor grade, (g) lymphovascular invasion, and (h) perineural invasion.

We next investigate the relationship between the risk score and clinical parameters in the TCGA database. Only OSCC samples with complete clinical information (including patient tumor stage and grade (including N and T stages), genders, age, and the presence of perineural and lymphovascular invasion) were used. We found that, in addition to age (Figure 5(a)), perineural and lymphovascular invasion were found to correlate significantly with the hypoxia-immune mRNA signature, but not with patient tumor stage and grade (including N and T stages) and genders (Figures 5(b)–5(h)).

Both univariate and multivariate analyses were done on the TCGA dataset to further prove the significance of the constructed hypoxia- and immune-related gene signatures. The risk score, age, and pathological stage may be able to be combined to predict the prognosis of the TCGA OSCC cohort (Table 2).

Finally, we investigated whether our model could be used to determine survival outcomes in subgroups with different clinicopathological features. Supplementary Figures 2A–2I show that the risk score could be used to predict the prognosis of patients with different clinicopathological features.

### 3.5. Variability of Degree of Immune Cell Infiltration between the Low- and High-Risk TCGA OSCC Cohorts

**Figure 6:**
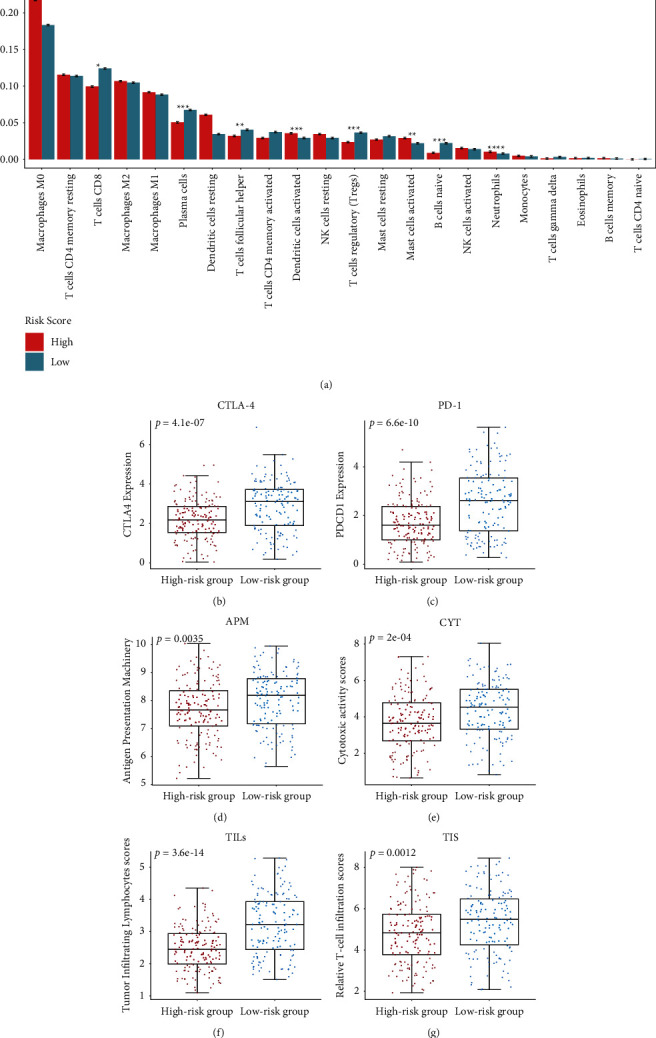
Analysis of immune status variability of the high- and low-risk groups in the TCGA OSCC cohort. (a) The bar plot shows various immune cell infiltration between the high- and low-risk patients. (b-g) Box plots illustrating markedly different immune checkpoints between the high- and low-risk groups.

The degree of immune cell infiltration between low- and high-risk groups was evaluated using the CIBERSORT algorithm. Those in the low-risk group were more likely to have higher infiltration degrees of CD8+ T cells, plasma cells, follicular helper T cells, regulatory T cells (Tregs), and naive B cells. On the other hand, high-risk patients demonstrated higher degrees of infiltration of activated DCs, activated mast cells, and neutrophils. Furthermore, the immune indicator scores and the immune checkpoint expressions in the low-risk group were all remarkably raised in comparison to those in the high-risk group patients (Figures 6(b)–6(g)).

To further assess the ability of our model to predict immunotherapy efficacy, the IMvigor210 cohort of MIBC patients treated with PD-L1 inhibitors was used. The Kaplan–Meier analysis showed that patients with high-risk scores had a poorer survival rate than those with low-risk scores (Supplementary Figure 3A, *p*=0.018). ROC curve analyses showed that the risk score combined with tumor mutational burden (TMB) and tumor neoantigen burden (TNB) output a higher area under the curve (AUC) value (AUC = 0.699) than TMB (AUC = 0.659), TNB (AUC = 0.690), or risk score (AUC = 0.560), respectively (Supplementary Figure 3B). Furthermore, we further discussed the differences in immunotherapy response between the high-risk group and the low-risk group based on the immune signature, and the results found that patients in the low-risk group had a higher complete response (CR)/partial response (PR) rate than those in the high-risk group (Supplementary Figure 3C).

## 4. Discussion

OSCC is the most common oral cancer characterized by a higher recurrence rate and lower overall survival rate of patients [11]. Hypoxia has been found to play an important role in the prognosis and treatment of OSCC [28]. Hypoxia also influences the activity of immune cells in the tumor microenvironment [31]. For example, hypoxia can impair the maturation and activity of dendritic cells (DCs) and natural killer (NK) cells [31, 32]. Both hypoxia and immune status play important roles in OSCC [33–35]. However, there is a lack of a reliable prognostic model of integration of hypoxia- and immune-related signatures for OSCC. In this study, we have a comprehensive analysis and constructed a hypoxia-immune-integrated indicator in OSCC.

Based on hypoxia-related signatures, the samples in our study were grouped into 3 clusters (Cluster 1, Cluster 2, and Cluster 3) (Figures 1(a)–1(c)). The presence of cell hypoxia induces the expression of HIF-1*α*. Enhanced HIF-1*α* expression has previously been associated with poor prognosis and lymph nodes metastasis in OSCC patients [36]. Further evaluation of overall patient survival resulted in the combination of Clusters 2 and 3 to form the Hypoxia_High group, while those in Cluster 1 were designated as the Hypoxia_Low group (Figure 1(d)). The expression of genes from two hypoxia-related gene sets, “increased oxygen delivery” and “reduced oxygen consumption,” was compared between Cluster 1 and Clusters 2 and 3. Eleven of the 14 genes belonging to the “increased oxygen delivery” were expressed at higher levels in Clusters 2 and 3 in contrast to Cluster 1 (Figure 1(e)). On the other hand, 7 of the 12 genes associated with “reduced oxygen consumption” were highly expressed in Cluster 1 compared to Clusters 2 and 3 (Figure 1(f)). Therefore, Cluster 1 and Clusters 2 and 3 were considered as the Hypoxia_Low and Hypoxia_High groups, respectively. A total of 773 DEGs were identified across both these groups (Figures 1(e) and 1(f)).

We then divided these OSCC patients into three groups (Immune_High, Immune_Medium, and Immune_Low) according to the abundance of immune cells (Figure 2(a)). The immune score of Immune_High group was higher than that of the Immune_Medium and Immune_Low groups. The Immune_Low group has the lowest immune score among the three groups (Figures 2(b) and 2(c)). A total of 854 immune-related genes in OSCC were determined between the Immune_High and Immune_Low groups (Figures 2(e) and 2(f)). All genes were found to be enriched in immune-related functions, including “leukocyte activation involved immune response,” “adaptive immune response,” “lymphocyte activation,” and “immunoregulatory interactions between a Lymphoid and a non-Lymphoid cell” (Figure 2(f)). These immune processes are also related to the malignant properties of OSCC. Previous reports found that a higher neutrophil-to-lymphocyte ratio was a negative predictor for overall survival for patients with OSCC [37]. Dendritic cell immune response activation was able to be induced by IFN-*γ-*inhibited OSCC growth in tumor-bearing mice [38].

To further integrate the hypoxia- and immune-related genes, the overlapping genes between the two gene sets were screened. Eight prominent mRNA signatures associated with OS from the 193 overlapping genes were selected, which were FAM122C, RNF157, RANBP17, SOWAHA, KIAA1211, RIPPLY2, INSL3, and DNAH1 (Figures 3(a)–3(d)). Among these mRNAs, KIAA1211 is known to be an oncogenic gene. Non-small-cell lung cancer tissues were found to have raised KIAA1211 expressions in contrast to adjacent normal tissues. Knockdown of KIAA1211 inhibited the proliferative abilities of NSCLC cells while promoting apoptosis both *in vitro* and *in vivo* [39]. Small cell lung cancer patients with a KIAA1211 mutation possess a longer survival period than those with wild-type KIAA1211 mutations [40]. RIPPLY2 represented one of the mRNAs in a five-gene signature verified to be able to predict the survival of endometrial cancer patients [41]. The tumor-promoting effect of INSL3 in cancer has also been widely addressed. The plasma level of INSL3 was found to be raised in an individual with metastatic ovarian cancer [42]. Other reports highlight the potential role of INSL3 as a marker of human testicular Leydig cell tumors [43]. INSL3 could promote tumor growth and angiogenesis in nude mice model of thyroid cancer in a manner that appeared to be related to the action of RXFP2 and the secretion of S100A4 and (pro-) cathepsin-L [44]. In pancreatic cancer patients, a higher serum level of INSL3 was associated with increased anorexia [45]. These mRNAs, which have previously been found to be of significant value in other cancers, should be further investigated for their role in OSCC.

These 8 mRNAs were used to construct an mRNA signature, which may have prognostic potential in OSCC (Figures 3(e)–3(h)). This constructed risk score was then validated in a cohort from the GEO dataset (Figures 4(a)–4(f)). The risk score showed a significant association only with age and perineural and lymphovascular invasion, but not with other features, including gender, T stage, N stage, and tumor stage (Figures 5(a)–5(h)). Therefore, this established gene signature may be an independent prognostic indicator.

Cancer immunotherapy has obtained much attention in recent years and is considered as a direction of tumor therapy, such as checkpoint blocking therapy. The specific difference in the tumor immune environment can reflect the heterogeneity of clinical samples in response to current immunotherapy. Finally, the immune profile variability between the high- and low-risk groups was compared in the TCGA OSCC cohort. High-risk group samples showed different degree of immune cell infiltration (Figure 6(a)). The low-risk group showed higher immune status than the low-risk group, as evidenced by a higher amount of immune checkpoint expression, including CTLA-4, PD-1, APM, CYT, and TILs (Figures 6(b)–6(g)). These checkpoints are indictors of OSCC risk and may function as therapeutic targets in OSCC. The genetic variants of CTLA-4 were associated with tobacco-related OSCC risk in the North Indian population [46]. A high number of CTLA-4 cells is associated with poor 5-year metastasis-free survival of OSCC patients [47]. An anti-PD-1 antibody is an agent which may prevent the initiation and progression of OSCC while prolonging patient survival time [48–50]. Analysis of the IMvigor210 cohort revealed that patients with low-risk scores had better survival and tended to have a higher complete response (CR)/partial response (PR) rate.

In conclusion, hypoxia and immune status play a key role in the prognosis of OSCC. Combining hypoxia- and immune-related genes, we established an OSCC prognostic model based on hypoxia and immunity, which provides a reliable reference for clinical decision-making.

A preprint has previously been published on the preprint website (https://www.researchsquare.com/article/rs-596220/v1) [51].

## Figures and Tables

**Table 1:** Clinical characteristics of the TCGA OSCC patients.

Characteristic	High (*n* = 161)	Low (*n* = 142)	TCGA (*n* = 303)
*Age*			
≤62	80	81	161
>62	81	61	142

*Gender*			
Female	51	48	99
Male	110	94	204

*Survival status*			
Living	93	100	193
Dead	68	42	110

*Pathologic M*			
Unknown	100	88	188
M0	61	54	115

*Pathologic N*			
Unknown	27	17	44
N0	59	54	113
N1	27	19	46
N2	47	51	98
N3	1	1	2

*Pathologic T*			
Unknown	11	7	18
T1	12	15	27
T2	50	43	93
T3	31	26	57
T4	57	51	108

*Tumor stage*			
Unknown	13	10	23
Stage I	9	8	17
Stage II	27	24	51
Stage III	33	21	54
Stage IV	79	79	158

**Table 2:** Univariate and multivariate Cox regression analysis of clinicopathological features associated with overall survival in TCGA data.

Variables	Univariate analysis		Multivariate analysis	
	HR (95% CI)	*P*-value	HR (95% CI)	*P*-value
Age (＞62/≤62)	1.021 (1.005–1.037)	0.00942^*∗*^	1.0329 (1.0103–1.056)	0.00413^*∗*^
Gender (male/female)	1.021 (0.6894–1.511)	0.919	0.7058 (0.4183–1.191)	0.19194
Tumor grade (G4/G3/G2/G1)	1.35 (1.01–1.803)	0.0424^*∗*^	1.4226 (0.9819–2.061)	0.06242
Stage (IV/III/II/I)	1.482 (1.172–1.874)	0.00102^*∗*^	1.7513 (1.0060–3.049)	0.04758^*∗*^
Pathologic T (T4/T3/T2/T1)	1.447 (1.181–1.773)	0.000365^*∗*^	1.0449 (0.7235–1.509)	0.81483
Pathologic N (N3/N2/N1/N0)	1.411 (1.116–1.785)	0.00403^*∗*^	1.1601 (0.8620–1.561)	0.32721
Risk score	4.322 (2.096–8.912)	0.0000736^*∗*^	3.5246 (1.4818–8.383)	0.00438^*∗*^

*Note.* ^*∗*^Statistically significant. HR: hazard ratio; CI: confidence interval.

## Data Availability

The data used are obtained from TCGA Data Portal: https://portal.gdc.cancer.gov/, GEO Datasets: https://www.ncbi.nlm.nih.gov/gds/, and Metascape: https://metascape.org/.

## Supplementary Materials

Supplementary Materials
*Supplementary Figure 1.* The expression of the eight signature genes. (A) Differential expression profiles of the 8 signature genes in low and high-risk groups. (B) Differential expression profiles of the 8 signature genes in low and high-risk groups. *Supplementary Figure 2.* Kaplan–Meier analyses of the eight-gene-based model in subgroups with different clinicopathological features. Kaplan–Meier survival curves for (A) age >62, (B) age ≤62, (C) male, (D) female, (E) T1 + T2, (F) T3 + T4, (G) N0 + N1, (H) N2 + N3, (I) stage I + stage II, (J) stage III + stage IV, (K) G1 + G2, and (L) G3 for the low- and high-risk groups. T: stage-T, N: stage-N, M: stage-M, and G: tumor grade. *Supplementary Figure 3*. The risk model predicts immunotherapy efficacy. (A) Kaplan–Meier overall survival curves for patients assigned to low- and high-risk groups in the IMvigor210 cohort. (B) ROC curves of tumor mutational burden (TMB), tumor neoantigen burden (TNB), risk score, and the combination (TMB, TNB, and risk score). (C) Rate of clinical response (CR/PR and SD/PD) to immunotherapy in the low- or high-risk groups (CR, complete response; PR, partial response; PD, progressive disease; SD, stable disease).”
