# Supplementary figures and images for: Identification and Validation of a Hypoxia-Immune-Based Prognostic mRNA Signature for Oral Squamous Cell Carcinoma
Source: J Oncol. 2022 Feb 7;2022:5286251. doi: 10.1155/2022/5286251 (PMC8844353; doi:10.1155/2022/5286251)

**A**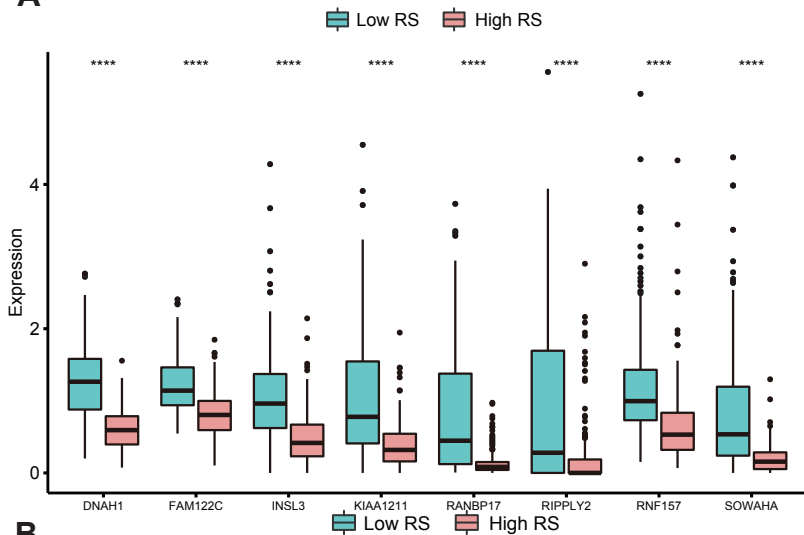**B**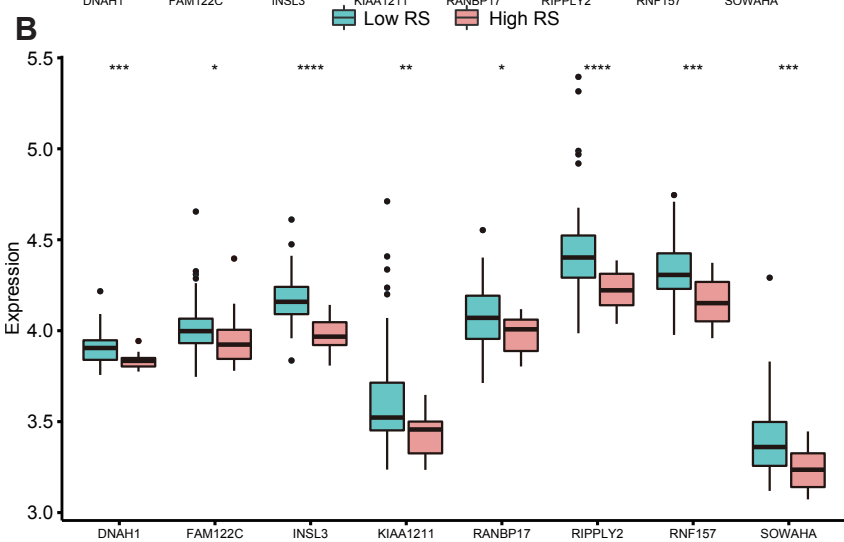

Supplement: Supplementary Materials — Supplementary Figure 1. The expression of the eight signature genes. (A) Differential expression profiles of the 8 signature genes in low and high-risk groups. (B) Differential expression profiles of the 8 signature genes in low and high-risk groups. Supplementary Figure 2. Kaplan–Meier analyses of the eight-gene-based model in subgroups with different clinicopathological features. Kaplan–Meier survival curves for (A) age >62, (B) age ≤62, (C) male, (D) female, (E) T1 + T2, (F) T3 + T4, (G) N0 + N1, (H) N2 + N3, (I) stage I + stage II, (J) stage III + stage IV, (K) G1 + G2, and (L) G3 for the low- and high-risk groups. T: stage-T, N: stage-N, M: stage-M, and G: tumor grade. Supplementary Figure 3. The risk model predicts immunotherapy efficacy. (A) Kaplan–Meier overall survival curves for patients assigned to low- and high-risk groups in the IMvigor210 cohort. (B) ROC curves of tumor mutational burden (TMB), tumor neoantigen burden (TNB), risk score, and the combination (TMB, TNB, and risk score). (C) Rate of clinical response (CR/PR and SD/PD) to immunotherapy in the low- or high-risk groups (CR, complete response; PR, partial response; PD, progressive disease; SD, stable disease).” [file 5286251.f1.zip › 5286251.f1/supplementary Figure 1.pdf]

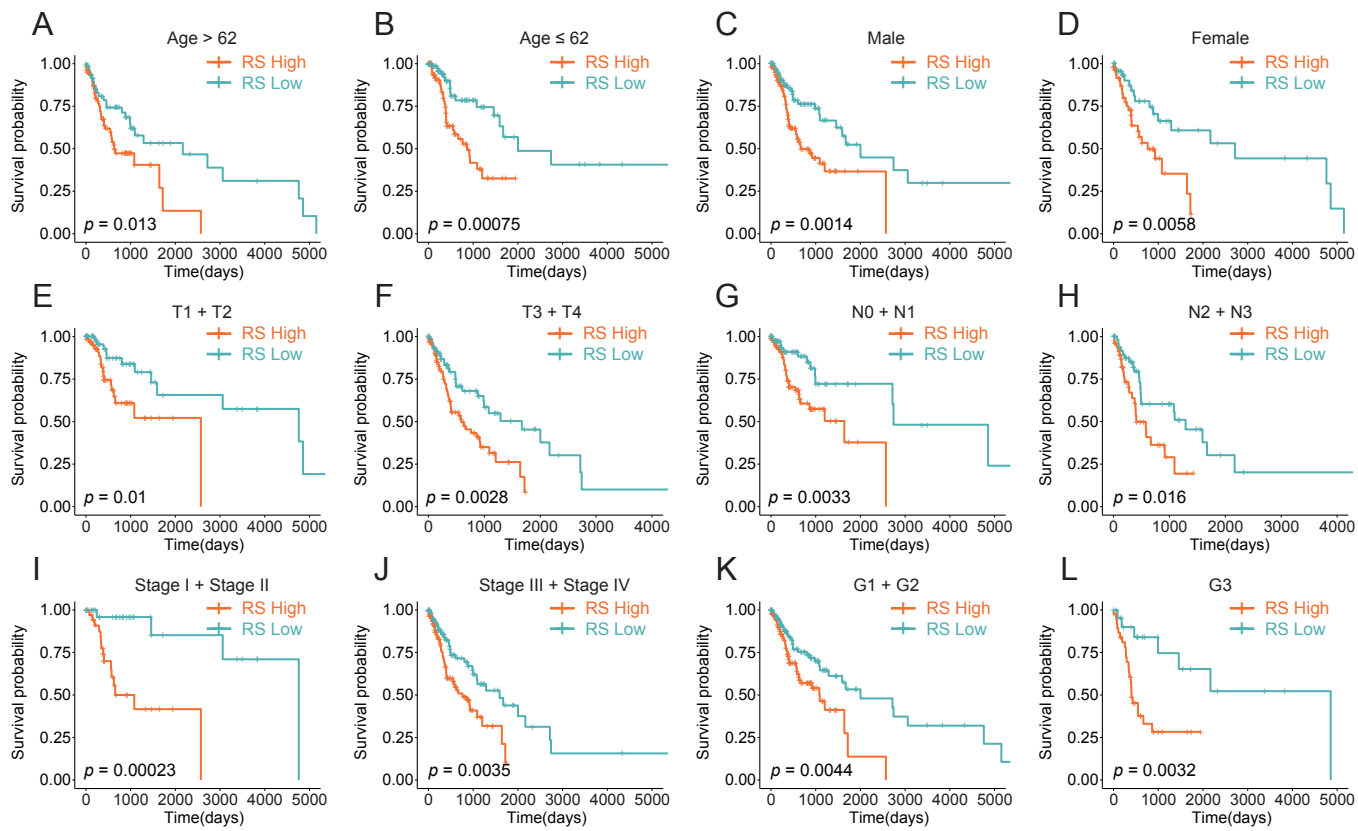

Supplement: Supplementary Materials — Supplementary Figure 1. The expression of the eight signature genes. (A) Differential expression profiles of the 8 signature genes in low and high-risk groups. (B) Differential expression profiles of the 8 signature genes in low and high-risk groups. Supplementary Figure 2. Kaplan–Meier analyses of the eight-gene-based model in subgroups with different clinicopathological features. Kaplan–Meier survival curves for (A) age >62, (B) age ≤62, (C) male, (D) female, (E) T1 + T2, (F) T3 + T4, (G) N0 + N1, (H) N2 + N3, (I) stage I + stage II, (J) stage III + stage IV, (K) G1 + G2, and (L) G3 for the low- and high-risk groups. T: stage-T, N: stage-N, M: stage-M, and G: tumor grade. Supplementary Figure 3. The risk model predicts immunotherapy efficacy. (A) Kaplan–Meier overall survival curves for patients assigned to low- and high-risk groups in the IMvigor210 cohort. (B) ROC curves of tumor mutational burden (TMB), tumor neoantigen burden (TNB), risk score, and the combination (TMB, TNB, and risk score). (C) Rate of clinical response (CR/PR and SD/PD) to immunotherapy in the low- or high-risk groups (CR, complete response; PR, partial response; PD, progressive disease; SD, stable disease).” [file 5286251.f1.zip › 5286251.f1/supplementary Figure 2.pdf]

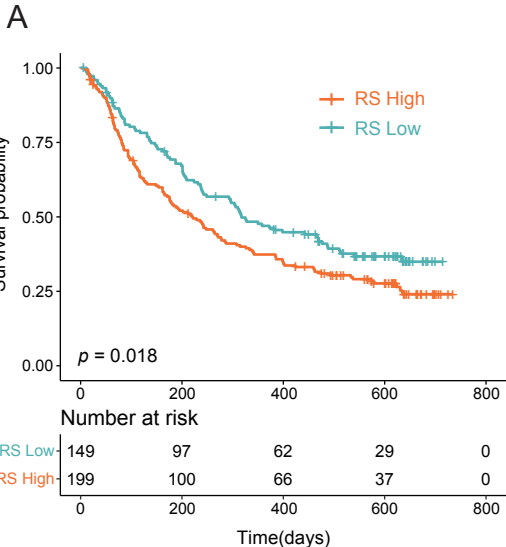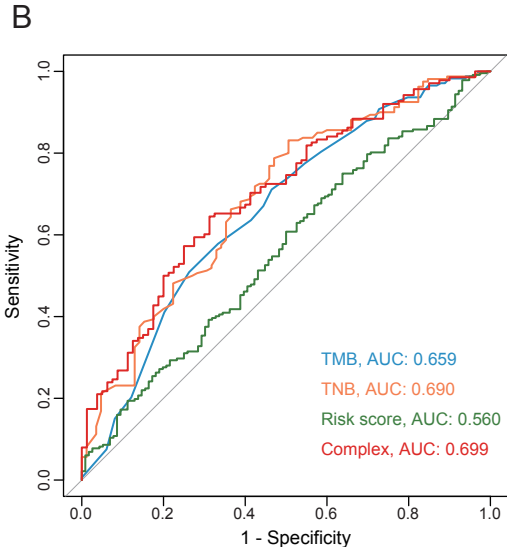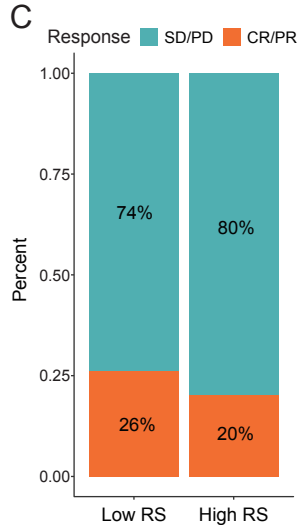

Supplement: Supplementary Materials — Supplementary Figure 1. The expression of the eight signature genes. (A) Differential expression profiles of the 8 signature genes in low and high-risk groups. (B) Differential expression profiles of the 8 signature genes in low and high-risk groups. Supplementary Figure 2. Kaplan–Meier analyses of the eight-gene-based model in subgroups with different clinicopathological features. Kaplan–Meier survival curves for (A) age >62, (B) age ≤62, (C) male, (D) female, (E) T1 + T2, (F) T3 + T4, (G) N0 + N1, (H) N2 + N3, (I) stage I + stage II, (J) stage III + stage IV, (K) G1 + G2, and (L) G3 for the low- and high-risk groups. T: stage-T, N: stage-N, M: stage-M, and G: tumor grade. Supplementary Figure 3. The risk model predicts immunotherapy efficacy. (A) Kaplan–Meier overall survival curves for patients assigned to low- and high-risk groups in the IMvigor210 cohort. (B) ROC curves of tumor mutational burden (TMB), tumor neoantigen burden (TNB), risk score, and the combination (TMB, TNB, and risk score). (C) Rate of clinical response (CR/PR and SD/PD) to immunotherapy in the low- or high-risk groups (CR, complete response; PR, partial response; PD, progressive disease; SD, stable disease).” [file 5286251.f1.zip › 5286251.f1/supplementary Figure 3.pdf]
